# What to Do with Non-visualized Sentinel Nodes? A Dutch Nationwide Survey Study

**DOI:** 10.1245/s10434-017-5824-4

**Published:** 2017-03-03

**Authors:** Nicole C. Verheuvel, Adri C. Voogd, Vivianne C. G. Tjan-Heijnen, Rudi M. H. Roumen

**Affiliations:** 1 Department of Surgery, Máxima Medical Center, Veldhoven, The Netherlands; 2 Department of Medical Oncology, School for Oncology and Developmental Biology (GROW), Maastricht University Medical Center, Maastricht, The Netherlands; 3 Department of Epidemiology, School for Oncology and Developmental Biology (GROW), Maastricht University Medical Center, Maastricht, The Netherlands; 4 Department of Research, Netherlands Comprehensive Cancer Organisation (IKNL), Utrecht, The Netherlands

## Abstract

**Introduction:**

International guidelines differ regarding their recommendations on axillary treatment of patients with non-visualized sentinel lymph nodes (non-vSLN). Therefore, we distributed a survey among Dutch oncological surgeons to determine their routine practice and opinion regarding axillary treatment in case of a non-vSLN, with the emphasis on whether these practices and opinions have changed since publication of the Z0011 trial.

**Methods:**

A Dutch nationwide survey containing 10 questions regarding clinical routine during the sentinel node procedure and axillary treatment of non-vSLN patients was distributed among 510 oncological surgeons.

**Results:**

The survey was completed by 122 (24%) oncological surgeons, of whom 116 (95%) were registered as specialized breast surgeons. These surgeons had, on average, 13 years of experience. The majority of respondents used both lymphoscintigraphy and Patent Blue during the sentinel node procedure, and 39% estimated the prevalence of a non-vSLN to be 1–2%. Most surgeons are currently more reserved when considering whether to perform an axillary lymph node dissection (ALND) than prior to publication of the Z0011 trial (15 vs. 80%, respectively). Sixty percent base their decision on various clinicopathological characteristics. Twenty-three respondents (20%) opted for an alternative axillary treatment.

**Conclusion:**

This study shows that, in daily practice, most specialized breast surgeons think that a non-vSLN is rare. If so, most currently opt not to perform an ALND, whereas a small proportion consider an alternative axillary treatment. These decisions differ than in the period prior to the Z0011 trial. More research is needed to provide optimal treatment recommendations in case of a non-vSLN.

**Electronic supplementary material:**

The online version of this article (doi:10.1245/s10434-017-5824-4) contains supplementary material, which is available to authorized users.

Since its introduction in the 1990s, the sentinel lymph node (SLN) procedure has become a standard element in the axillary work-up. Prior to introduction of the SLN procedure, every patient with invasive breast cancer underwent an axillary lymph node dissection (ALND). The SLN procedure has made it possible to select patients without axillary metastases, in whom the ALND could then be omitted. More recently, studies such as the Z0011 trial also showed that the ALND could be omitted in selected SLN-positive patients.^1^–^4^ However, in 2–4% of SLN procedures, the sentinel node cannot be visualized and retrieved (non-vSLN), after which an ALND should still be performed according to the current Dutch guideline.^5^–^7^

Scientific studies are scarce and international guidelines differ in their recommendations on whether or not an ALND should be performed in case of a non-vSLN.^6^,^8^–^10^ The lack of scientific evidence may lead to different axillary treatment regimens between hospitals and surgeons. Therefore, we distributed a survey among certified oncological surgeons in The Netherlands in order to gain some insight into their routines during the SLN procedure, and to gauge respondents’ opinions regarding axillary treatment options in case of a non-vSLN. Do they perform an immediate ALND in all patients with non-vSLN or do they differentiate between patients based on indicative factors? Did they change their routine practice after publication of the Z0011 trial?

## Methods

The survey was developed by the authors and distributed, through mail, among certified surgeons registered with the Dutch Association of Surgical Oncologists (NVCO), a subdivision of the Dutch Association of Surgery (NVVH). The survey was distributed among 510 oncological surgeons, of whom 116 were registered as specialized breast cancer surgeons. Because publication of the Z0011 trial caused a paradigm shift in the axillary management of node-positive patients, questions were designed to assess practice patterns both before and after publication of the Z0011 trial. The final question of the survey was to gauge the respondents’ perceptions on the current Dutch guideline regarding the axillary management of patients with a non-vSLN (see Supplementary Material Appendix 1 for the complete survey).

## Results

### Respondents

Of the 510 distributed surveys, a total of 122 (24%) were returned by surgical oncologists, which represented 95% of all registered specialized breast surgeons. The median number of years working as an oncological surgeon was 13, ranging from 1 to 35 years. Twenty-six (21%) respondents had up to 5 years’ experience as a surgeon, 24 (20%) had 5–10 years’ experience, 23 (19%) had 10–15 years’ experience, 18 (15%) had 25–20 years’ experience, 7 (6%) had 20–25 years’ experience, 10 (9%) had 25–30 years’ experience, and one respondent had over 30 years of experience.

### Sentinel Node Procedure

During the SLN procedure, 87% (106/122) reported using both the lymphoscintigraphy and Patent Blue technique, while 13% (16/122) used the lymphoscintigraphy technique only. None of the respondents used Patent Blue only. When asked to estimate how many times the sentinel node could not be visualized, 35% (43/122) answered <1%, 39% (48/122) answered between 1 and 2%, 25% (30/122) answered between 2 and 5%, and one respondent estimated the sentinel node could not be visualized in more than 5% of cases. The estimated prevalence of non-vSLNs was not significantly different between surgeons who only used the lymphoscintigraphy technique versus those who combined lymphoscintigraphy with Patent Blue. When asked to estimate the prevalence of any lymph node metastases in patients with a non-vSLN, 26% (30/117) of respondents estimated it to be <25%, 38% (44/117) estimated it to be between 25 and 30%, and 37% (43/117) estimated it to be more than 30%.

### Axillary Treatment

**Fig. 1:**
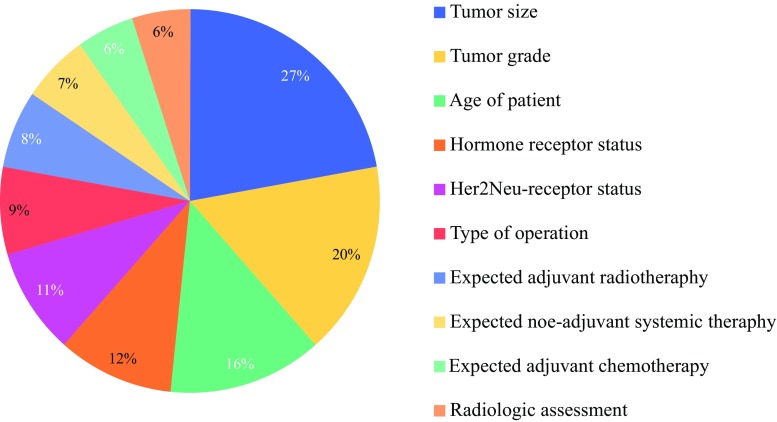
Clinicopathological factors influencing decision making on whether or not to perform an immediate ALND in case of a non-vSLN, with percentage of respondents mentioning these factors. *ALND* axillary lymph node dissection, *non*-*vSLN* non-visualized sentinel lymph node

Table 1 shows additional responses to the survey. What stands out is that in all scenarios of an unsuccessful SLN harvesting, neither with lymphoscintigraphy nor with Patent Blue, axillary treatment has changed substantially after publication of the Z0011 trial. Prior to the Z0011 trial, the majority (approximately 80%) of respondents answered they would always perform an ALND in case of a non-vSLN. After publication of the Z0011 trial, this number has dropped to approximately 15%. At present, the majority of respondents (60%) declared they only perform an ALND when they expect an increased risk of nodal involvement based on various clinicopathological characteristics, as illustrated in Fig. 1. In the past, only 10–15% of breast surgeons based their decision on such clinical factors. Moreover, 23 (19%) respondents answered they would opt for an alternative axillary treatment instead of a complete ALND in case of a non-vSLN; two respondents indicated they would not perform an ALND at all, 11 respondents indicated they would perform an incomplete ALND, seven respondents would opt for 4-node sampling, two respondents would redo the SLN procedure, and one respondent would consult a multidisciplinary team for further treatment recommendations.

**Table 1:** Responses on questions asked in the survey regarding routines of axillary treatment in case of a non-vSLN, prior to versus after the Z0011 trial

Questions		
	Prior to the Z0011 trial	Currently
*1 (A* + *B)*	*N* = 120	*N* = 122
What do/did you do when the sentinel node could not be visualized after lymphoscintigraphy and the use of a gamma probe?		
I will perform an immediate axillary lymph node dissection (ALND)	35 (30.3%)	1 (0.8%)
I will refrain from further axillary treatment	0	5 (4.1%)
I will attempt to find the sentinel node by means of Patent Blue (see question 2)	79 (64.8%)	92 (75.4%)
Whether I will perform an immediate ALND depends on patient and tumor characteristics, such as …	6 (4.9%)	22 (18%)
Never happened	0	2 (16%)
*2 (A* + *B)*	*N* = 115	*N* = 118
If in the previous question you chose to attempt to find the sentinel node by means of Patent Blue (option C), what do/did you do in case the sentinel node could not be visualized during this procedure?		
I will perform an immediate ALND	94 (81.7%)	17 (14.4%)
I will refrain from further axillary treatment	4 (3.5%)	27 (22.9%)
Whether I will perform an immediate ALND depends on patient and tumor characteristics, such as …	13 (11.3%)	71 (60.2%)
Never happened	4 (3.5%)	3 (2.5%)
*3 (A* + *B)*	*N* = 118	*N* = 119
What do/did you do in case the sentinel node could be visualized during the lymphoscintigraphy, but not during the operation, after using Patent Blue?		
I will perform an immediate ALND	94 (79.7%)	19 (16%)
I will refrain from further axillary treatment	1 (0.8)	25 (21%)
Whether I will perform an immediate ALND depends on patient and tumor characteristics, such as …	1 (14.4%)	67 (56.3%)
Never happened	6 (51%)	8 (6.7%)
	No	Yes
4		
I still execute the Dutch guideline of 2012 regarding the axillary work-up and treatment (*n* = 117)	63 (54%)	54 (46%)
Currently, the guideline regarding axillary work-up is clear-cut (*n* = 120)	85 (71%)	35 (29%)
I always perform a sentinel node procedure (*n* = 121)	11 (9%)	110 (91%)
Sometimes, in case of a negative axillary ultrasound, I omit further axillary diagnostics, including the sentinel node procedure (*n* = 120)	114 (95%)	6 (5%)
In addition to the axillary ultrasound, I apply additional imaging techniques to evaluate axillary nodal status, such as PET, PET/CT, MRI, etc. (*n* = 119)	63 (53%)	56 (47)
The confusion on the axillary work-up has increased (*n* = 120)	34 (28%)	86 (72%)
In my opinion, the sentinel node procedure will be obsolete and will disappear within the next few years (*n* = 120)	80 (67%)	40 (33%)
Surgical treatment of the axilla is, or will be, redundant (*n* = 119)	94 (79%)	25 (21%)
The guideline should be revised regarding further		
axillary treatment in case of a non-visualized sentinel node. If so, which aspect? (*n* = 113)	24 (21%)	89 (79%)

*non*-*vSLN* non-visualized sentinel lymph node, *PET* positron emission tomography, *CT* computed tomography, *MRI* magnetic resonance imaging

Subanalyses differentiating between years of experience as a surgeon (less than 10 vs. 10 or more years) showed no significant differences in the methodology of the SLN procedure or in preferences for axillary treatment in case of a non-vSLN, prior to or after the Z0011 trial. What did differ was that the experienced surgeons (10 or more years of experience) more often expected the ALND to be redundant in the future, and they thought that the guideline should be revised for patients with a non-vSLN.

### Additional Comments

At the end of the survey, respondents could add supplementary comments and opinions. The comment most frequently made was that an ALND should not be performed on patients with a low risk of axillary disease, depending on tumor characteristics and the age of the patient, as shown in Fig. 1. Some respondents recommended administering adjuvant axillary radiotherapy or performing a partial, instead of a complete, ALND, especially if there is an indication for adjuvant systemic therapy.

Many respondents indicated that the upcoming revised Dutch guideline regarding axillary treatment in case of a non-vSLN should not only differentiate between patients with low risk of axillary disease versus patients with high risk but should also be more specific on the best axillary treatment options, such as partial ALND or axillary radiotherapy.

## Discussion

The present study shows the results of a nationwide survey, which was distributed among certified oncological (breast) surgeons in The Netherlands, regarding routine practice during the SLN procedure and the axillary treatment in case of a non-visualized and non-retrieved SLN. The data show that respondents are currently more reserved in performing a complete ALND in case of a non-vSLN compared with the period prior to publication of the Z0011 trial. Moreover, the indication to perform an ALND nowadays mostly depends on clinicopathological factors.

The Z0011 trial caused a paradigm shift in the axillary treatment of clinically node-negative but SLN-positive patients. This trial compared the effects of ALND after SLNB versus SLNB alone on (disease-free) survival and concluded that omitting the ALND in some of these patients did not negatively affect (disease-free) survival. Patients in whom the ALND could be omitted can be selected by using the so-called ‘Z0011 criteria’: (i) invasive breast cancer; (ii) clinical tumor size ≤5 cm (T1–2); (iii) no palpable lymphadenopathy; (iv) one or two positive sentinel nodes; and (v) treated with lumpectomy.^1^ In addition, the European Organization for Research and Treatment of Cancer (EORTC) AMAROS (After Mapping of the Axilla: Radiotherapy or Surgery) trial showed that in SLN positive patients with T1–2 invasive breast cancer without lymphadenopathy, administering axillary radiotherapy was not inferior to an ALND in providing locoregional disease control.^2^ Next to these two studies, numerous other studies have shown that the ALND is no longer absolutely necessary in every breast cancer patient and, consequently, various prediction models and scoring systems have been developed to select patients in whom the ALND could be omitted.^11^–^15^ Although these studies provide a promising perspective for breast cancer patients, as the ALND can cause significant morbidity, they only included patients with a positive SLN, thereby disregarding clinically node-negative patients in whom the SLN could not be retrieved.

This scarcity of studies has also caused some ambiguity between international guidelines in axillary treatment recommendations in case of a non-vSLN, provided that treatment options are mentioned at all. The current Dutch NABON guideline states that in case of a non-vSLN, an ALND should be performed for locoregional control.^6^ The recommendations in the American Society of Clinical Oncology (ASCO) guideline, as well as the Australian guideline, are identical to the Dutch guideline. The National Comprehensive Cancer Network (NCCN) guideline also concurs; however, a footnote has been added which states that in case of clinically negative axillary lymph nodes and treatment with mastectomy and radiation therapy, an extended radiation field to the axilla may also be sufficient.^8^,^9^,^16^ Currently, only the NCCN guideline has implemented the Z0011 criteria in its recommendations.^1^,^9^

Both the European Society for Medical Oncology (ESMO) and British National Institute for Health and Care Excellence (NICE) guidelines do not mention the possibility of a non-vSLN, or its implications for treatment.^10^,^17^

Therefore, the question remains as to what the optimal axillary treatment is in case of a non-vSLN. Studies have shown that older age, a high body weight, larger tumor size, and a high number of positive lymph nodes and macrometastases can decrease the success rate of the SLN procedure.^5^,^18^–^23^ Therefore, perioperative palpation of the axilla remains important, especially in case of a non-vSLN, to locate any suspicious nodes that are not identified by Patent Blue or lymphoscintigraphy. Although we did not explicitly ask this in our survey, in The Netherlands this is common practice. In a previous study, which has yet to be published, we examined potential differences in patient characteristics and prognosis between patients with a non-vSLN versus those in whom SLN was successfully harvested (vSLN). This study showed that older patients, patients diagnosed prior to 2006, and patients with a large tumor were more likely to have an unsuccessful SLN procedure. Moreover, non-vSLN patients had a significantly worse survival compared with patients in whom the SLN could be retrieved. However, performing an ALND in non-vSLN patients did not significantly improve prognosis. This supports the tendency of many surgeons to not (unconditionally) perform a complete ALND in case of a non-vSLN.

The present survey is the first to assess current clinical practice among Dutch oncological surgeons in case of a non-vSLN. Although this survey is limited and not validated yet, its high response by Dutch breast surgeons makes it quite representative. The results provide relevant and important insights into the diversity in axillary treatment given by Dutch breast surgeons in case of a non-vSLN. Moreover, this survey has shown that decisions on whether or not to perform an ALND in case of a non-vSLN has changed after publication of the Z0011 trial, irrespective of the surgeon’s years of experience. A more extensive and validated international survey is warranted for more scientifically-based conclusions on the diversity of given axillary treatments.

## Conclusions

The results of this survey show that Dutch breast surgeons are more reserved to perform an ALND in patients with a non-visualized sentinel node, especially after publication of the Z0011 trial. According to the answers given in this survey, the decision on whether or not to perform an ALND nowadays mostly depends on multiple clinicopathological characteristics. More research is warranted to determine the optimal axillary treatment in patients with non-vSLN to be able to provide evidence-based recommendations in international guidelines.

## Electronic supplementary material

Below is the link to the electronic supplementary material.

Supplementary material 1 (DOCX 23 kb)
