# Supplementary material for: What to Do with Non-visualized Sentinel Nodes? A Dutch Nationwide Survey Study
Source: Ann Surg Oncol. 2017 Mar 3;24(8):2155–60. doi: 10.1245/s10434-017-5824-4 (PMC5491635; doi:10.1245/s10434-017-5824-4)
Supplement: Supplementary file 1 — Supplementary material 1 (DOCX 23 kb) [file 10434_2017_5824_MOESM1_ESM.docx]

# Appendix

**Appendix 1: Survey on non-visualized sentinel nodes**

Introduction

- Since ……. years I work as a (oncological) surgeon.
- During the sentinel lymph node procedure you use:
  - Lymphoscintigraphy
  - Patent Blue
  - Combination of both
- The average chance of having a positive sentinel node depends on multiple factors, especially primary tumor characteristics, but also on pre-operative work-up such as a sonographic evaluation of the axilla. Generally, it has been estimated that the chance of having a positive sentinel node is between 25 and 30%.

What is your estimation on the chance of having 1 or more positive axillary lymph nodes in case the sentinel node could not be visualized?

- - I estimate this chance to be between 25 and 30%
  - This chance is smaller
  - This chance is higher
- I estimate that the prevalence of a non-visualized sentinel node is:
  - Less than 1%
  - Between 1 and 2%
  - Between 2 and 5%
  - More than 5%

**Below there are 5 questions regarding the axillary work-up 5 to 10 years ago, prior to publication of the Z0011 trial and the AMAROS trial?**

***1-A*** What did you do when the sentinel node could not be visualized after lymphoscintigraphy and the use of a gamma probe?

1. I would have performed an immediate axillary lymph node dissection (ALND)
2. I would have omitted further axillary treatment
3. I would have attempted to find the sentinel node by means of Patent Blue
4. Whether I would have performed an immediate ALND depended on patient and tumor characteristics, such as…………………………………………………………

***2-A*** If in the previous question you chose to attempt to find the sentinel node by means of Patent Blue (option c), what did you do in case the sentinel node could not be visualized during this procedure?

1. I would have performed an immediate axillary lymph node dissection (ALND)
2. I would have omitted further axillary treatment
3. Whether I would have performed an immediate ALND depended on patient and tumor characteristics, such as…………………………………………………………

***3-A*** What did you do in case the sentinel node could be visualized during the lymphoscintigraphy, but not during the operation after using Patent Blue?

1. I would have performed an immediate axillary lymph node dissection (ALND)
2. I would have omitted further axillary treatment
3. Whether I would have performed an immediate ALND depended on patient and tumor characteristics, such as…………………………………………………………

**Please answer the same questions, however, now regarding the axillary work-up *at this moment***

***1-B*** What did you do when the sentinel node could not be visualized after lymphoscintigraphy and the use of a gamma probe?

1. I will perform an immediate axillary lymph node dissection (ALND)
2. I will omit further axillary treatment
3. I will attempt to find the sentinel node by means of Patent Blue
4. Whether I will perform an immediate ALND depends on patient and tumor characteristics, such as.………………………………………………………………

***2-B*** If in the previous question you chose to attempt to find the sentinel node by means of Patent Blue (option c), what did you do in case the sentinel node could not be visualized during this procedure?

1. I will perform an immediate axillary lymph node dissection (ALND)
2. I will omit further axillary treatment
3. Whether I will perform an immediate ALND depends on patient and tumor characteristics, such as.………………………………………………………………

***3-B*** What did you do in case the sentinel node could be visualized during the lymphoscintigraphy, but not during the operation after using Patent Blue?

1. I will perform an immediate axillary lymph node dissection (ALND)
2. I will omit further axillary treatment
3. Whether I will perform an immediate ALND depends on patient and tumor characteristics, such as.………………………………………………………………
4. Please indicate whether you agree with the following statements. Multiple answers are possible.

In 2015:

| - - I still execute the Dutch guideline of 2012 regarding the axillary work-up and treatment. | Yes / No |
| --- | --- |
| - - Currently, the guideline regarding axillary work-up is clear-cut. | Yes / No |
| - - I always perform a sentinel node procedure. | Yes / No |
| - - Sometimes, in case of a negative axillary ultrasound I omit further axillary diagnostics, including the sentinel node procedure | Yes / No |
| - - In addition to the axillary ultrasound, I apply additional imaging entities to evaluate axillary nodal status, such as PET, PET/CT, MRI, etc. | Yes / No |
| - - The confusion on the axillary work-up has increased. | Yes / No |
| - - In my opinion, the sentinel node procedure will be obsolete and will disappear within the next few years. | Yes / No |
| - - Surgical treatment of the axilla is or will be redundant. | Yes / No |
| - - The guideline should be revised regarding further axillary treatment in case of a non-visualized sentinel node.   If so, which aspect? ……. | Yes / No |

Additional comments:

|  |
| --- |

**References**

1. NABON national guideline breast cancer 2.0; Comprehensive Cancer Centre Netherlands (2012). www.oncoline.nl/mammacarcinoom.
2. National Comprehensive Cancer Network. NCCN clinical practice guidelines in oncology (NCCN guidelines 2014); breast cancer version 1.2014. www.nccn.com.
3. National Breast Cancer Centre (NBCC). Recommendations for use of sentinel node biopsy in early (operable) breast cancer (2008). Canceraustralia.gov.au
